# Research on the Corrosion Behavior of Q235 Pipeline Steel in an Atmospheric Environment through Experiment

**DOI:** 10.3390/ma15186502

**Published:** 2022-09-19

**Authors:** Shuo Cai, Hongchao Ji, Fengyun Zhu, Weichi Pei, Wenchao Xiao, Xuefeng Tang

**Affiliations:** 1 College of Mechanical Engineering, North China University of Science and Technology, Tangshan 063210, China; 2 CHINA 22 MCC GROUP CORPORATION LIMITED, Tangshan 063035, China; 3 School of Engineering and Technology, China University of Geosciences (Beijing), Beijing 100083, China; 4 State Key Laboratory of Materials Processing and Die & Mould Technology, Huazhong University of Science and Technology, 1037 Luoyu Road, Wuhan 430074, China

**Keywords:** Q235 low-carbon steel, corrosion, cyclic salt spray test, corrosion behavior mechanism, rust product morphology

## Abstract

Low-carbon steel pipelines are frequently used as transport pipelines for various media. As the pipeline transport industry continues to develop in extreme directions, such as high efficiency, long life, and large pipe diameters, the issue of pipeline reliability is becoming increasingly prominent. This study selected Q235 steel, a typical material for low-carbon steel pipelines, as the research object. In accordance with the pipeline service environment and the accelerated corrosion environment test spectrum, cyclic salt spray accelerated corrosion tests that simulated the effects of the marine atmosphere were designed and implemented. Corrosion properties, such as corrosion weight loss, morphology, and product composition of samples with different cycles, were characterized through appearance inspection, scanning electron microscopy analysis, and energy spectrum analysis. The corrosion behavior and mechanism of Q235 low-carbon steel in the enhanced corrosion environment were studied, and the corrosion weight loss kinetics of Q235 steel was verified to conform to the power function law. During the corrosion process, the passivation film on the surface of the low-carbon steel and the dense and stable α-FeOOH layer formed after the passivation film was peeled off played a role in corrosion resistance. The passivation effect, service life, and service limit of Q235 steel were studied and determined, and an evaluation model for quick evaluation of the corrosion life of Q235 low-carbon steel was established. This work provides technical support to improve the life and reliability of low-carbon steel pipelines. It also offers a theoretical basis for further research on the similitude and relevance of cyclic salt spray accelerated corrosion testing.

## 1. Introduction

With the development of the petroleum, metallurgical, mechanical, and chemical industries in the 20th century, pipeline transportation has entered a stage of rapid development thanks to its powerful advantages, such as large transport volume, low cost, safety, and reliability. It is now widely used in various industrial installations, such as urban domestic water supply, long-distance transport of oil and gas, agricultural irrigation, and machine tool hydraulics [1,2,3]. Q235 low-carbon steel is used in a large number of transport pipelines thanks to its high processability, low cost, and good forming ability [4,5,6]. However, pipeline failure has long been regarded as a fundamental constraint on the development of the pipeline transportation industry, with pipeline corrosion being one of the major causes of pipeline failure [7,8,9]. The European Gas Pipeline Incident Data Organization (EGIG) reported 1366 pipeline incidents among EGIG member countries and organizations between 1970 and 2016. The percentage of pipeline accidents caused by pipeline corrosion reaches as high as 15.09%, and liquid pipelines are two to three times more likely to fail than gas pipelines [10,11,12]. The global economic cost of pipeline corrosion can reach as much as US$2.2 trillion per year, or about 3% of the world’s gross domestic product [13,14]. Therefore, the corrosion behavior and damage laws of pipelines, particularly Q235 low-carbon steel pipelines, in various natural atmospheric and working environments have elicited increasing attention from experts, scholars, and engineers. A series of studies have been conducted on the service life and limit of pipelines in service and indoor simulation environments.

The atmospheric corrosion of low-carbon steel is controlled by type and environmental conditions, such as wetting time, air pollutants, temperature, and dust content [15,16]. Xiao et al. explored the corrosion products and formation mechanism of carbon steel during the early stage of atmospheric corrosion; they used scanning electron microscopy (SEM) and Raman spectroscopy to observe the ring-shaped and chain-shaped corrosion products formed on the surface of carbon steel during the early stage of corrosion [17]. Mendoza et al. compared the outdoor and indoor atmospheric corrosion of carbon steel; they determined the metrological parameters of major pollutants, such as chloride ions and SO_2_, and then proposed and developed a model to represent the relationship between the corrosion of carbon steel and environmental parameters [18,19]. By contrast, the corrosion products formed in the atmosphere of carbon steel are frequently protective, thus the corrosion rate decreases with time. Almeida et al. presented new insights into the role of carbon dioxide in the corrosion mechanism of carbon steel, demonstrating that no direct reaction of carbon dioxide occurs on free iron surfaces [20]. Dugstad and Li et al. suggested that the corrosion rate of carbon steel in a CO_2_ environment is largely dependent on the formation of protective corrosion product films; moreover, controlling influences, such as pH and temperature, can promote the precipitation of corrosion products and reduce the overall corrosion rate [21,22].

Compared with other types of atmospheric environment, the marine atmospheric environment is more typical. Refait et al. investigated the role of corrosion product layers in the corrosion of carbon steel in the marine environment, focusing on the mechanism of the formation of the first solid phase, i.e., sulfate green rust, precipitated from the dissolved material produced by corrosion on the steel surface. The relationship between the current coupling between the anodic and cathodic regions of a metal surface and the heterogeneous corrosion product layer during the localized corrosion process was also discussed [23]. Ma et al. demonstrated via loss-in-weight analysis that the mechanism and kinetics of low-carbon steel during atmospheric corrosion exhibit a transitional behavior in a marine environment with a high chloride ion content and relative humidity, with the average corrosion rate influenced by Cl^−^ ions reaching the maximum amount during the transition period and then fluctuating within a certain range [24,25]. Song et al. firmly believed that relative humidity exerts a significant effect on the corrosion of Q235 carbon steel under a thin electrolyte layer in marine atmosphere, with higher relative levels of α-FeOOH and lower relative levels of β-FeOOH in the corrosion products in high humidity environments [26]. Yu et al. studied the exposure corrosion of Q235 carbon steel and Q450 weathering steel in a hot and dry environment; their analysis showed that the major components of the steel surface’s rust layer were FeOOH, Fe_3_O_4_, Fe_2_O_3_, and other iron-rich oxides [27].

In addition, the corrosion rate is an important parameter for the corrosion of carbon steel. Slaimana et al. studied the corrosion rate of carbon steel pipes under turbulent conditions; they concluded that, as corrosion products form and surface roughness increases, the coefficient of friction on carbon steel surfaces also increases with the temperature, Reynolds number, and corrosion rate [28,29]. Clover et al. found that the corrosion rate of carbon steel pipes is influenced by microstructure, and they concluded that a relationship exists between local corrosion susceptibility and the pearlite zone in steel microstructure, whereas no significant correlation exists between the trace element concentration and corrosion resistance [30]. Although numerous experimental data and research are available on the corrosion behavior and rate of carbon steel in atmospheric environment, low-carbon steel pipes must be pickled and passivated before they are used in actual production. Therefore, when studying the corrosion process of Q235 low-carbon steel in an atmospheric environment, including the surface passivation film in the analysis scope is highly significant for exploring the corrosion behavior and mechanism of Q235 low-carbon steel and determining the service life.

At present, natural environmental exposure testing is still the basic method for studying and evaluating the service life and corrosion resistance of metallic materials. However, most metal parts are generally designed to last for more than 10 years, thus directly conducting routine exposure experiments that simulate the natural environment of service is costly and inefficient in terms of time and money [31,32,33]. Therefore, manually accelerated tests under laboratory conditions can produce valid data within a short period of time and are suitable for assessing the corrosion resistance and service life of metals in certain specific atmospheric environments [34,35,36]. The neutral salt spray (NSS) test is an environmental test that uses artificially simulated salt spray environmental conditions created by salt spray test equipment to assess the corrosion resistance of products or metal materials. Its principle lies in simulating coastal atmospheric conditions, i.e., warm sea surface evaporates from the cold air and tiny droplets that contain chloride ions splash into space under the impact of waves, forming the corrosion conditions of fine mist sprayed onto metal. The use of a certain concentration of sodium chloride solution, sprayed under pressure with fine mist, is basically in line with the basic principles of atmospheric corrosion, because mist particles fall uniformly onto the surface of the passivation layer and constantly maintain the renewal of the liquid film. Given its low economic and time costs, NSS is frequently used in verifying the service environment and duration of medium-sized pipelines [37,38,39]. The cyclic salt spray accelerated corrosion test used in the current study is an improved test based on the NSS test, and its fitting degree to the environment can exceed 0.9. In addition, the corrosion behavior and mechanism of the passivated Q235 low-carbon steel were comprehensively characterized in terms of corrosion kinetics, corrosion product morphology and composition, and corrosion rate via corrosion weight loss, SEM, and energy-dispersive X-ray spectroscopy (EDS). It provides a theoretical basis for further research on the similitude and relevance of accelerated corrosion testing and exhibits practical significance for the safe and efficient operation of Q235 low-carbon steel pipelines in actual production.

## 2. Experiment Procedures

**Figure 1:**
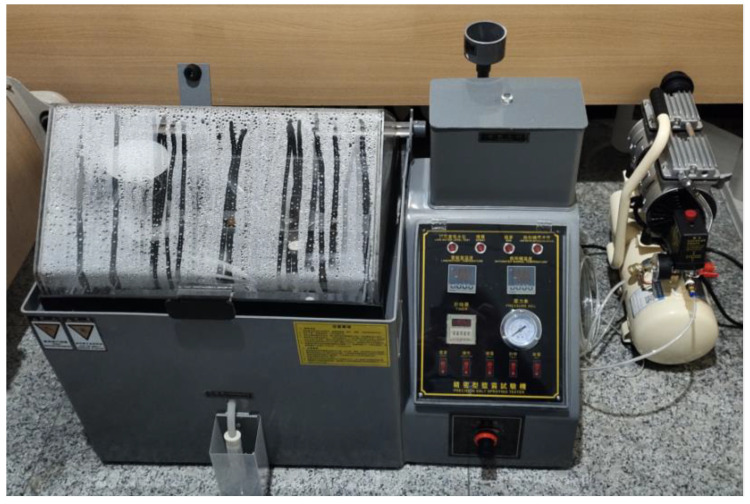
Salt spray tester with air compressor.

The samples used for testing were passivated Q235 low-carbon steel with the chemical composition provided in Table 1. The sample production process and test procedure were as follows: the Q235 low carbon steel pipe was cut into 15 mm × 20 mm × 2 mm samples with a wire cutting machine, and the method of cyclic cleaning was adopted to ensure the uniformity of pickling and passivation treatment. The sample was placed in the pipeline cleaning tank, and the temperature was 42 °C for the pickling solution (its main components are hydrochloric acid, oxalic acid, and urotropine corrosion inhibitor) in the tank for 3.8 h of pickling treatment. After neutralizing the pickling solution, the passivation solution at 25 °C (the main component of which is a mixed solution of sodium nitrite and a passivator accelerator) was passed into the pipeline cleaning tank for 0.5 h passivation treatment. After passivation, the surface of the qualified sample should be free of rust and evenly covered with a dense dark black passivation film. The treated specimens were numbered, dried, and weighed on an electronic scale, with the results achieving an accuracy of 0.1 mg. The weighed test pieces were placed in a salt spray tester, as shown in Figure 1.

The accelerated corrosion method used salt spray dry and wet cycles. The test medium was 5 wt% NaCl solution. The pH in the salt spray chamber was controlled within the range of 6.5–7.2. The average settling rate of the horizontal area of 80 cm^2^ was 1.5 mL/h ± 0.5 mL/h. The spray temperature was (40 ± 1) °C for 4 h. The drying temperature was (60 ± 1) °C for 4 h. The cycle time was 8 h, while the dry and wet transformation time was less than 0.5 h.

During the test, the samples were subjected to corrosion damage in strict compliance with the specified test conditions and application sequence. Sampling and corrosion performance were tested at the test nodes at 4, 8, 12, 16, and 20 days. The test duration was 20 days. The number of parallel samples in each group was one piece, and two pieces were reserved for the original sample, i.e., a total of 2 × 5 + 2 = 12 pieces.

Appearance inspection: The samples were periodically removed and placed in an indoor environment to dry naturally for 1 h before being carefully cleaned with running water at a temperature of less than 40 °C to remove any residual salt from the surface. Drying treatment with a dry hot air stream followed before macroscopic morphological observation and analysis were performed.

Corrosion weight loss analysis: A configured special rust remover was used to eliminate corrosion products on the surface of a sample in accordance with the method provided in GB/T 16545-2015. The sample was rinsed with water, soaked in anhydrous ethanol, removed, dried immediately using dry hot air, and placed on an electronic scale for weighing and recording sample weight loss data. The corrosion kinetics law and variation equation of carbon steel can be obtained through mass comparison before and after the corrosion of the samples and the fitting of the weight loss curve. The atmospheric corrosion kinetics of metals follows the power function law, i.e.,

(1) $$D=At^{n},$$

where D is the corrosion weight loss (or depth), mm; t is the exposure time, years; A is the corrosion rate in the first year; and n is a constant—the smaller its value, the better the corrosion resistance of carbon steel. The corrosion weight loss and corrosion rate curves were plotted, and corrosion rate was calculated in accordance with the following formula:

(2) $$R=\frac{(w_{0}-w_{t})\times 10^{4}}{S\rho t},$$

where R is the corrosion weight loss rate, μm/d; $w_{0}$ and $w_{t}$ are the mass of the specimen before and after corrosion, g; S is the exposed area of the specimen, cm^2^; ρ is the density of Q235 steel, 7.86 g/cm^2^; and t is the exposure time of the specimen in salt spray, days.

Analysis of corrosion morphology and corrosion product composition: SEM was used to observe and analyze the microscopic morphology of a sample’s surface during each stage of the experiment. It was combined with EDS to analyze the chemical composition of the corrosion products qualitatively and explore the growth process and the corrosion mechanism of corrosion products on the surface of Q235 low-carbon steel.

## 3. Results and Discussion

### 3.1. Analysis of the Macroscopic and Micro-Morphological Changes of the Sample

**Figure 2:**
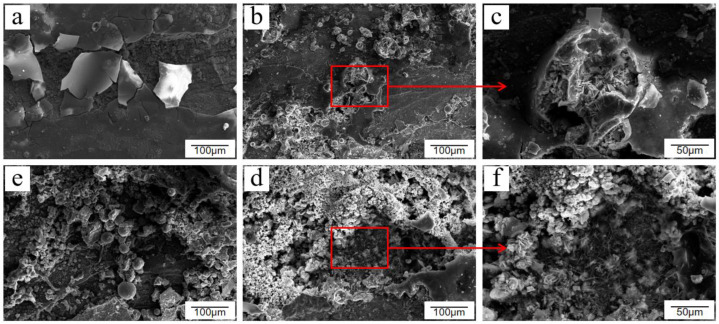
Several corrosion product morphologies of Q235 low-carbon steel under atmospheric conditions: (**a**) membranous, (**b**) needlelike, (**c**) needle enlargement, (**d**) crystal cluster, (**e**) crystal-like, and (**f**) crystal enlargement.

In general, the corrosion of Q235 low-carbon steel without passivation treatment in the atmospheric environment is divided into three stages. The first stage is surface hydroxylation, in which a thin layer of oxide or hydroxyl oxide is formed on the surface of carbon steel within a short period. The second stage is when the atmosphere acts as a thin liquid film attached onto the surface of the tube wall, in which its constituents are dissolved, leading to the transformation of the thin layer of oxides or hydroxyl oxides into green rust. The third stage is the gradual increase in the number and size of the nuclei of the products and the transformation of green rust into a yellow-brown friable layer of oxides and hydroxides; thus, the corrosion products consist mostly of α-FeOOH, γ-FeOOH, and γ-Fe_2_O_3_. The corrosion product morphology is divided into three types: membranous, crystal-like, and crystal cluster-like, as shown in Figure 2a,d,e, respectively. Goethite (α-FeOOH) and lepidocrocite (γ-FeOOH) occasionally appear in the form of fibrous or needle-like aggregates during the corrosion process. These aggregates grow and rupture to form the three aforementioned forms of corrosion products. The corrosion products on the surface of Q235 low-carbon steel were randomly scattered and distributed with the corrosion degree and corrosion depth, as shown in Figure 2b,c,f.

**Figure 3:**
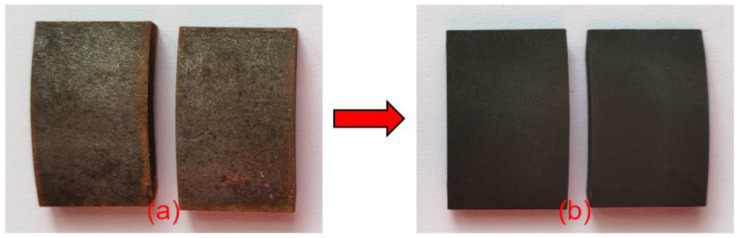
Comparison of samples before and after passivation: (**a**) before passivation and (**b**) after passivation.

**Figure 4:**
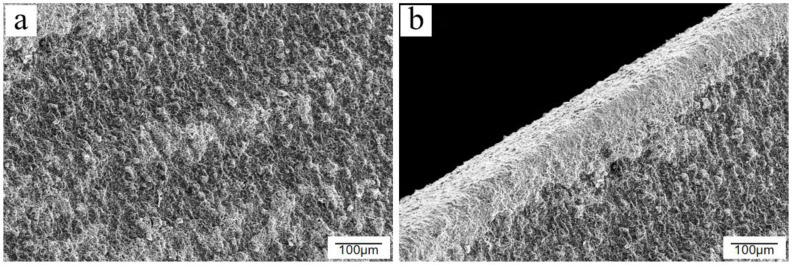
Comparison of passivation effects on different regions of the sample surface: (**a**) flat passivated layer and (**b**) passivated layer at fracture.

The vast majority of metals will corrode spontaneously in the general environment. The purpose of passivation is to form a uniform and dense oxide film on a metal’s surface via treatment with strong oxidants, reducing the surface activity of a metal to enhance its corrosion resistance. Before a metal is passivated, a pickling process is generally performed. After the pickling passivation process, surface rust marks on a test piece disappeared and the metallic silver-white surface was gradually covered by a black metal oxide, forming a dense oxide film, as shown in Figure 3. The observation of the passivated samples under an electron microscope revealed that large areas of flat and dense metal oxides replaced the original Q235 steel morphology, with a small number of raised or defective areas adversely affecting the formation of the passivated film, as shown in Figure 4a. There is also enough passivation product at the fracture, but compared with the passivation layer at the flat part, the color of the passivation layer at the fracture is lighter. This shows that the passivation product is affected by the ridges at the fracture, and the aggregation is relatively fluffy, which also lays the groundwork for the first rupture of the passivation film at the fracture in the subsequent salt spray experiment, as shown in Figure 4b. In various industries, multiple standards have been established for the thickness of passivation films on low-carbon steel, but generally, a maximum of 10 µm should not be exceeded.

When studying the corrosion behavior of metals in a “contaminated” atmosphere, applying the atmospheric corrosion model developed by Tomashov [40] in 1964 is common. On the basis of different liquid film thicknesses, this model divides the corrosion process into four categories: (1) when liquid film thickness is less than 10 nm, the process is dry atmospheric corrosion; (2) when liquid film thickness is 10 nm–1 µm, the process is humid atmospheric corrosion; (3) when liquid film thickness is 1 µm–1 mm, the process is wetting corrosion; and (4) when liquid film thickness is greater than 1 mm, the process is complete immersion corrosion. Therefore, the thin liquid film corrosion in the cyclic salt spray accelerated corrosion test is humid atmospheric corrosion, which is electrochemical in nature. 

**Figure 5:**
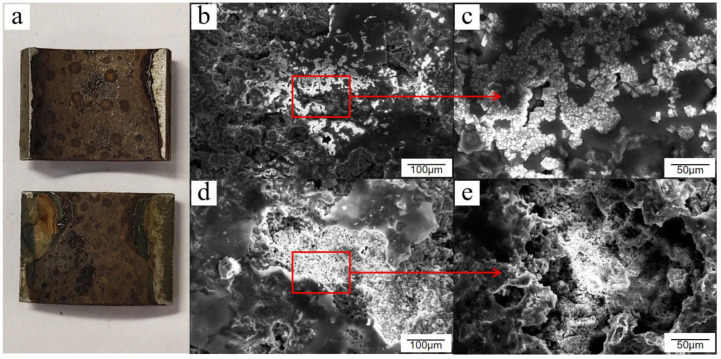
Macroscopic and microscopic morphology of Q235 low-carbon steel after 4 days of exposure in the cyclic salt spray accelerated corrosion test: (**a**) macroscopic appearance, (**b**) rupture area, (**c**) precipitation of crystals, (**d**) weak area, and (**e**) deep rupture.

Macroscopic and microscopic morphological changes on the surface of the Q235 low-carbon steel samples after exposure at different times in the cyclic salt spray accelerated corrosion test exhibited the following pattern. At the start of the test, the sodium chloride solution atomized by the salt spray tower was evenly dispersed over the surface of the carbon steel, forming an extremely thin liquid film that wrapped and covered the entire sample, continuously exerting a corrosive effect on the surface of the carbon steel. After 4 days, the black passivation film on the surface of Q235 low-carbon steel began to lighten in color and rust spots appeared. The corrosion process began transitioning from a passivation layer to a substrate rust layer. The passivation film broke at the fracture, revealing the original substrate form. From a microscopic point of view, the passivation film was not yet broken in the vast majority of the areas and still had some resistance to corrosion. However, some areas exhibited serious corrosion features, such as etch pits and rust spots, which were scattered and distributed. After magnification, the rupture residue of the passivation film and the newly generated corrosion products in the corrosion pits were mixed together and could not be easily distinguished. The corrosion depth was large. In addition, crystalline behavior was observed within the vicinity of the rust spots and pits, which consisted mostly of sodium chloride crystals that remained after cleaning. Combined with atmospheric moisture, the residual sodium chloride crystals will continue to cause damage to the passivation film, as shown in Figure 5.

**Figure 6:**
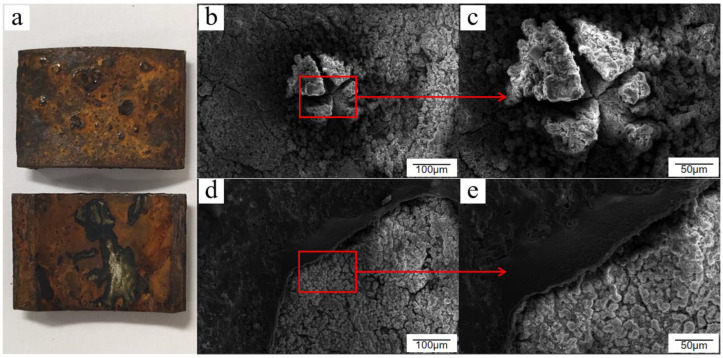
Macroscopic and microscopic morphology of Q235 low-carbon steel after 8 days of exposure in the cyclic salt spray accelerated corrosion test: (**a**) macroscopic appearance, (**b**) ruptured bulge, (**c**) enlarged drawing, (**d**) cracked edge, and (**e**) enlarged drawing.

From a macroscopic point of view, the majority of the black passivation film on the surface of Q235 low-carbon steel was replaced with a yellow-brown rust layer after 8 days of salt spray corrosion. The passivation film fell off over a large area, and the traces of passivation treatment were difficult to see, with local areas of granular corrosion products and the bulging phenomenon. From a microscopic point of view, the overall surface morphology of Q235 low-carbon steel was in the stage of passivation film breakage and shedding, with the growth of new rust products. Passivation film rupture was largely due to the combination of the passivation film residues and the new growth of rust products, constituting a mixed rust layer. Chloride ions and other corrosive media occurred under the loosening of cracking. Then, the corrosion product continued to grow and bulge, causing the passivation film to fall off, as shown by the bulge at the edge of the ruptured passivation film. In addition, the passivation film rupture area was littered with crystal clusters of corrosion products that were rich in needle-like morphological surface, which is the major feature of Q235 low-carbon steel surface micromorphology during this stage, as shown in Figure 6.

**Figure 7:**
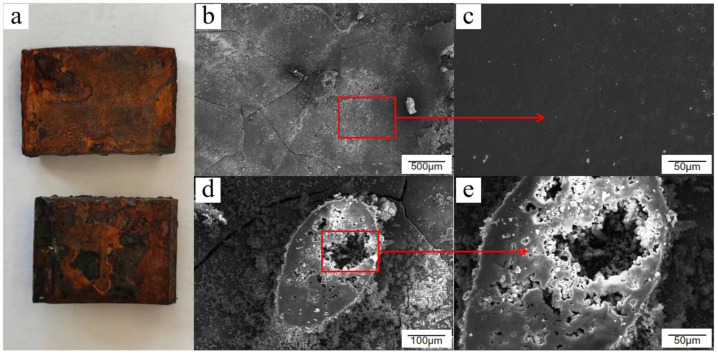
Macroscopic and microscopic morphology of Q235 low-carbon steel after 12 days of exposure in the cyclic salt spray accelerated corrosion test: (**a**) macroscopic appearance, (**b**) overall appearance, (**c**) flat rust layer, (**d**) rust cracked protrusion, and (**e**) internal corrosion growth.

The rust layer on the surface of Q235 low-carbon steel was not necessarily uneven and disorganized. By the time the salt spray test reached 12 days, the surface of the sample piece was completely replaced with a brownish rust product, which was darker in color compared with other periods. Its surface was flatter, with only small areas of slight defects. From a microscopic point of view, the residual passivation film was completely peeled off, and rust products were regenerated and extended onto the entire surface. A second flat corrosion layer was formed below the passivation film after stacking and agglomeration. This corrosion layer was denser than the other scattered rust traces. From the viewpoint of film formation of protective corrosion products, the corrosion layer is presumed as a “second protective layer” and, to a certain extent, it played a role in retarding corrosion and reducing the corrosion rate. However, the reduced corrosion rate does not mean that corrosion will not occur. Chloride ions and oxygen atoms will still penetrate and break through the corrosion layer and continue to exert a corrosive effect on the carbon steel substrate. The accumulation of newly generated corrosion products within the corrosion layer will again break through the corrosion layer and cause the surface to bulge and crack, as shown in Figure 7.

**Figure 8:**
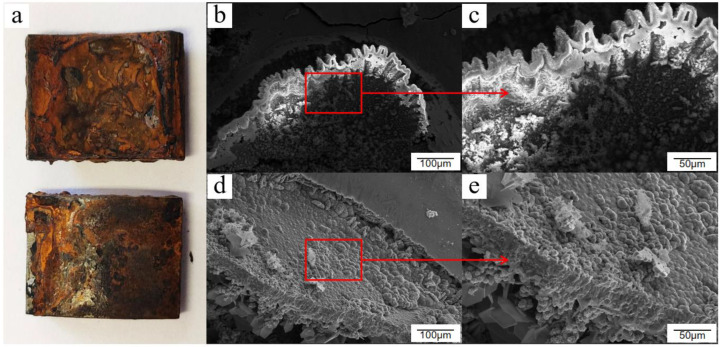
Macroscopic and microscopic morphology of Q235 low-carbon steel after 16 days of exposure in the cyclic salt spray accelerated corrosion test: (**a**) macroscopic appearance, (**b**) corrosion layer folds, (**c**) enlarged drawing, (**d**) corrosion product stacking up, and (**e**) enlarged drawing.

With the prolongation of exposure time, i.e., after the samples were placed in the salt spray environment for 16 days, large areas of folds and bulges appeared on the originally relatively flat rust layer. The color of the rust layer gradually deepened, and its thickness gradually increased, making the surface rust layer uneven in appearance. Using an electron microscope to magnify the characteristic area, the original flat carbon steel surface outside the corrosion layer was found to be either broken or covered, with various forms of corrosion products competing for growth. In the process of stacking and cascading, it continued to rise to a certain height after the fracture and the formation of “coral-like” folds. Considering that the protective layer formed in the previous phase is on the verge of falling off and failing, the corrosion rate is presumed to continue increasing, as shown in Figure 8.

**Figure 9:**
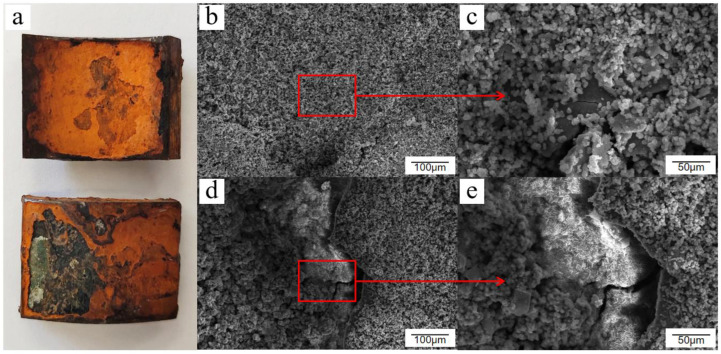
Macroscopic and microscopic morphology of Q235 low-carbon steel after 20 days of exposure in the cyclic salt spray accelerated corrosion test: (**a**) macroscopic appearance, (**b**) overall appearance, (**c**) loose corrosion products, (**d**) corrosion layer crack, and (**e**) corrosion intensifies.

After 20 days of cyclic salt spray accelerated corrosion testing, the surface rust products of Q235 low-carbon steel continued to accumulate and thicken until the rust layer began to fall off in a large area. The exposed base material under the erosion of chloride ions and other corrosive media continued to generate new corrosion products. The rust layer and substrate interface exhibited more evident delamination cracks. From a microscopic point of view, the surface of Q235 low-carbon steel was completely covered by layers of newly generated thin and loose crystal cluster corrosion products, as shown by the corrosion layer joints where new needle-like rusting materials were still being generated. These materials were first stacked and connected to form a metal oxide skin, and then continuously stacked on top of this skin to form various types of corrosion products, as shown in Figure 9.

Given the interaction of a variety of environmental factors, the current salt spray test is not a standard time conversion ratio. Predicting the salt spray environment can only be based on the Q235 steel pipe accelerated corrosion environmental test spectrum, in which 24 h is equal to 1 year in the natural environment. In various industries, the expected 20-year service life of carbon steel pipeline has been reached. Continuing the test will not be too meaningful, thus the test was stopped.

In summary, the corrosion of Q235 low-carbon steel in an atmospheric environment after pickling and passivation treatment originates from the weakness of the passivation film and gradually develops from pitting to full-scale corrosion, which is a “from point to surface” corrosion process. The whole process can be roughly divided into four stages: (1) passivation film corrosion product generation and local shedding, (2) matrix rust layer generation and covering, (3) rust layer accumulation and stacking onto a flat and dense second protective layer, and (4) matrix rust layer breaking through the protective layer continues to grow thicker until shedding.

### 3.2. Analysis of Changes in the Atomic Content of Corrosion Products

**Figure 10:**
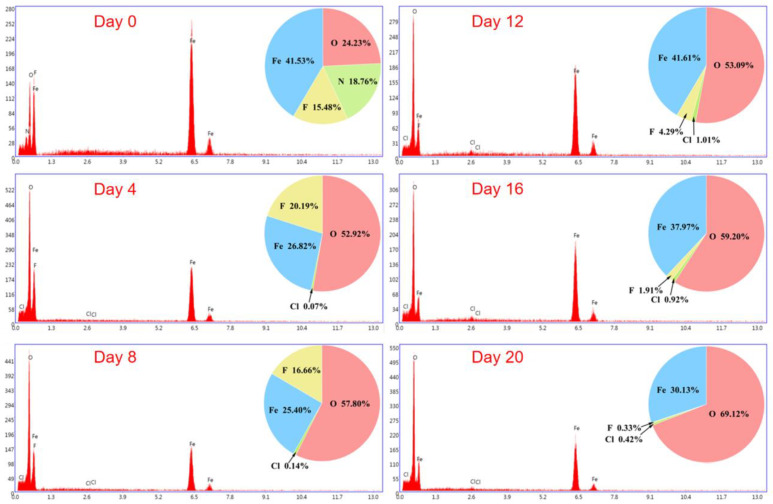
EDS spectra of the corrosion products of Q235 low-carbon steel after exposure to accelerated corrosive environments at different times.

**Figure 11:**
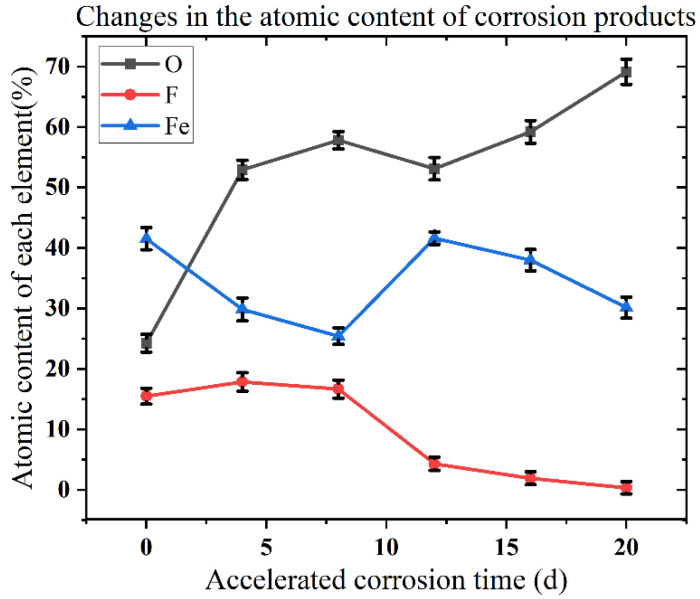
Changes in the elemental content of the corrosion products of Q235 low-carbon steel after different exposure times in accelerated corrosion environments.

The EDS energy spectrum of corrosion products and the change in the atomic content of each element with exposure time are shown in Figure 10 and Figure 11, respectively. Before the experiment started, because the surface of the Q235 low-carbon steel was covered with a uniform and dense passivation film, the content of nitrogen and fluorine atoms was relatively high. As the corrosion product of the passivation layer transitioned to the corrosion product of the base rust layer during the corrosion process, the passivation film began to corrode when the Q235 low-carbon steel sample was exposed to the salt spray environment for 0–4 days. Rust spots appeared on the surface, but the whole was not completely fractured, thus the proportion of fluorine atoms was relatively high. At 4–8 days of exposure, the passivation film on the sample surface was gradually covered by substrate rust. In some areas, the passivation film fell off with the substrate rust products, the fluorine atom content began to decrease, while the oxygen atoms began to increase. When exposed for 8–12 days, the residual passivation film was nearly completely removed, and a flat, dense matrix rust layer was formed. The fluorine atom content was significantly reduced, and the atomic fraction of iron oxides rose gradually with the thickening of the rust layer. When exposed for 12–16 days, the flat corrosion layer produced in the previous stage exerted a certain protective effect on the carbon steel substrate. Therefore, although the surface rust layer gradually deepened and thickened and the oxygen atom content increased, the rate of increase was relatively slow. At 16–20 days of exposure, the oxygen atom content rose considerably while the fluorine atom content decreased as the “second protective layer” failed and corrosion products continued to deepen and thicken. The reason for this phenomenon may be related to the local shedding of the corrosion layer after it thickened to a certain height, while the fluorine atom content was nearly negligible. Overall, the fluorine atom content tended to decrease from the start of the salt spray test and decreased at the fastest rate between 8 and 12 days, indicating that the passivation film exhibited an accelerated trend in the process of “from point to surface” rupture. Simultaneously, the oxygen atom content tended to increase, and then decrease, and then increase again, indicating that the passivation film on the surface of Q235 low-carbon steel and the “second protective layer” that formed after the passivation film broke down were fulfilling their expected protective role. In addition, the corrosion products contained a proportion of chlorine atoms, and this condition was mostly related to the deposition of the sprayed salt solution during the test.

The above analysis is consistent with the corrosion process of Q235 steel in the previous experiments. The composition of Q235 low-carbon steel rust layer after pickling and passivation treatment gradually transitions from the passivation product formed by the combination of nitric acid, hydrofluoric acid, and other oxidizing acids with carbon steel to iron oxide compounds mainly composed of ferric oxyhydroxide. 

### 3.3. Analysis of Corrosion Weight Loss

**Figure 12:**
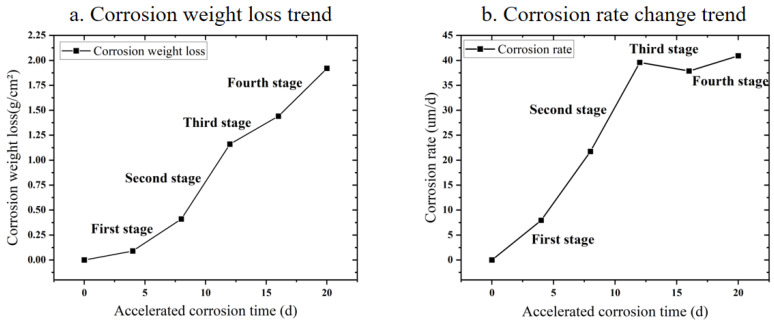
Corrosion kinetic analysis of Q235 low-carbon steel samples in an accelerated corrosive environment.

The corrosion weight loss and corrosion rate data of Q235 low carbon steel exposed to accelerated corrosion environment for different times are shown in Figure 12. By comparing the analysis of the data, the accelerated corrosion exposure process of Q235 low-carbon steel after passivation can be broadly divided into four stages. In stage 1 (0–8 days), corrosion weight loss tends to rise slowly and the corrosion rate increases at a slower rate. This scenario indicates that the passivation film forms a better protective effect on the substrate as the first corrosion-resistant layer during the early stage of corrosion. This phenomenon is related to the electrochemical protection mechanism of the passivation film. In the second stage (8–12 days), corrosion weight loss and the corrosion rate rapidly increased, demonstrating that, with the prolongation of exposure time, the substrate surface passivation film experienced comprehensive corrosion rupture. The loose rust layer was gradually peeled off, losing its protective effect. The substrate surface was exposed to hydrogen ions, chlorine ions, and other corrosive media. Electrochemical reaction resistance was rapidly reduced. In the third stage (12–16 days), the increasing trend of corrosion weight loss slowed down slightly, while the corrosion rate began to decline. This scenario was largely related to the gradual thickening of the rust layer and the increase in the proportion of stable α-FeOOH components. The formation of the second corrosion-resistant layer after the passivation film gradually reduced corrosive media and substrate contact area. The electrochemical reaction resistance increased. In the fourth stage (16–20 days), corrosion weight loss accelerated and the corrosion rate increased again, indicating that the carbon steel matrix was eroded as the more stable α-FeOOH rust layer thickened and fell off. At this moment, Q235 low-carbon steel had nearly no corrosion resistance.

**Figure 13:**
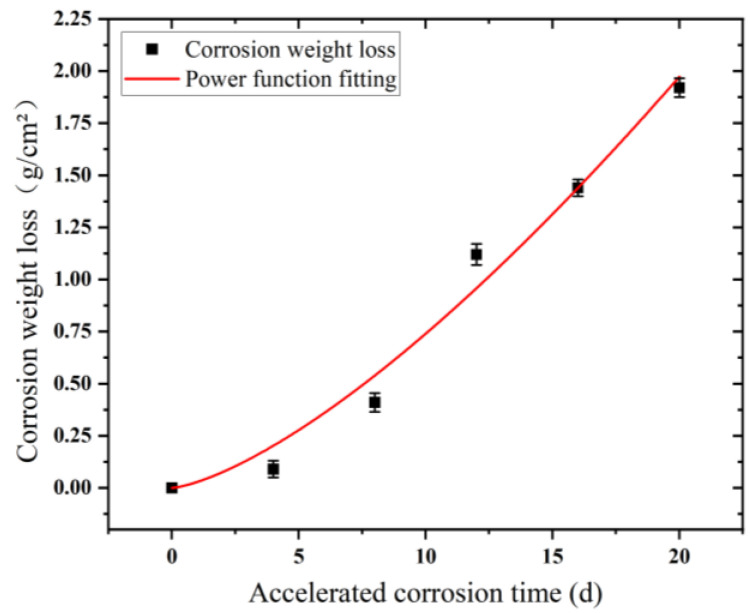
Corrosion weight loss fitting curves of Q235 low-carbon steel samples in accelerated corrosive environments.

In accordance with the trend of corrosion weight loss of Q235 low-carbon steel, the corrosion weight loss trend was fitted using Equation (1). The fitted curve and shape parameters are presented in Table 2 and Figure 13, respectively. Corrosion weight loss conformed to the power function change relationship, and the fitting degree reached as high as 0.976.

Q235 low-carbon steel has an *n* value greater than 1 during exposure, indicating a harsh environment for accelerated corrosion. Overall, Q235 low-carbon steel exhibits an accelerating corrosion process. The passivation film provides a good corrosion protective function during the early stage of corrosion. However, during the entire corrosion process, the passivation film falls off during the initial stage, and the surface rust layer on the substrate forms a protective effect, but is relatively weak. In the late stages of corrosion, the protective effect gradually appears. With the prolonged exposure time, the corrosion of Q235 low-carbon steel is likely to reach a relatively stable and slow stage, that is, the corrosion weight loss exponent n decreases below 1.

The above analysis verifies the four stages of the accelerated corrosion process of the Q235 steel atmospheric environment. The corrosion weight loss kinetics conforms to the power function change law, and the passivation film on the surface of Q235 low-carbon steel and the matrix rust layer formed after the passivation film rupture have had a good anti-corrosion effect.

## 4. Conclusions

In this study, the corrosion behavior and mechanism of Q235 in atmospheric environment are studied by designing a cyclic salt spray accelerated corrosion experiment. The specific conclusions are as follows:

(1) Q235 low-carbon steel in the atmospheric environment of the accelerated corrosion process can be roughly divided into four stages: (1) passivation film corrosion product generation and local shedding, (2) matrix rust layer generation and covering, (3) rust layer accumulation and stacking onto a flat and dense second protective layer, and (4) matrix rust layer breaking through the protective layer continues to grow thicker until shedding.
(2) After pickling passivation treatment of Q235 low-carbon steel in the atmospheric environment of corrosion from passivation film weakness, pitting corrosion gradually develops into comprehensive corrosion with the elongation of time. Rust layer composition gradually transitions from passivation products into iron oxide based on hydroxyl iron oxide. The whole process is “from point to surface” corrosion.
(3) Q235 low-carbon steel corrosion weight loss kinetics is in line with the power function law. The corrosion rate exhibits an increasing, decreasing, and then increasing trend, indicating that Q235 low-carbon steel surface passivation film and passivation film rupture formed after the matrix rust layer play a certain anti-corrosive role.

The results provide a theoretical basis for further research on the simulation and correlation of the cyclic salt spray accelerated corrosion test. It is verified that the service life of the Q235 low-carbon steel pipe has reached the 20-year period specified in the industrial standard, which has great practical significance for the application of the Q235 steel pipe in actual production.

## Figures and Tables

**Table 1:** Mass fraction of Q235 chemical composition.

Steel Model	Chemical Element Content (%)				
	C	Si	Mn	P	S
Q235A	0.22	0.35	1.40	0.045	0.050
Q235B	0.20	0.35	1.40	0.045	0.045

**Table 2:** Corrosion weight loss fitting results of Q235 low-carbon steel samples exposed to accelerated corrosive environments.

Exposure Period	Fitting Method	A	*n*	*R* ^2^
4–20 days	Power function	0.028	1.416	0.976
